# S1-Type Endonuclease 2 in Dedifferentiating *Arabidopsis* Protoplasts: Translocation to the Nucleus in Senescing Protoplasts Is Associated with De-Glycosylation

**DOI:** 10.1371/journal.pone.0170067

**Published:** 2017-01-09

**Authors:** Yemima Givaty-Rapp, Narendra Singh Yadav, Asif Khan, Gideon Grafi

**Affiliations:** French Associates Institute of Agriculture and Biotechnology of Drylands, The Institutes for Desert Research, Ben-Gurion University of the Negev, Midreshet Ben-Gurion, Israel; Stony Brook University, UNITED STATES

## Abstract

Cell dedifferentiation characterizes the transition of leaf cells to protoplasts and is accompanied by global chromatin decondensation. Here we show that in *Arabidopsis*, chromocentric chromatin undergoes prompt and gradual decondensation upon protoplasting. We hypothesized that prompt chromatin decondensation is unlikely to be driven solely by epigenetic means and other factors might be involved. We investigated the possibility that S1-type endonucleases are involved in prompt chromatin decondensation *via* their capability to target and cleave unpaired regions within superhelical DNA, leading to chromatin relaxation. We showed that the expression and activity of the S1-type endonuclease 2 (ENDO2) is upregulated in dedifferentiating protoplasts concomitantly with chromatin decondensation. Mutation of the *ENDO2* gene did not block or delay chromocentric chromatin decondensation upon protoplasting. Further study showed that ENDO2 subcellular localization is essentially cytoplasmic (endoplasmic reticulum-associated) in healthy cells, but often localized to the nucleus and in senescing/dying cells it was associated with fragmented nuclei. Using in gel nuclease assays we identified two ENDO2 variants, designated N1 (cytoplasmic variant) and N2 (cytoplasmic and nuclear variant), and based on their capability to bind concanavalin A (ConA), they appear to be glycosylated and de-glycosylated (or decorated with ConA non-binding sugars), respectively. Our data showed that the genome is responding promptly to acute stress (protoplasting) by acquiring decondensation state, which is not dependent on ENDO2 activity. ENDO2 undergoes de-glycosylation and translocation to the nucleus where it is involved in early stages of cell death probably by introducing double strand DNA breaks into superhelical DNA leading to local chromatin relaxation and fragmentation of nuclei.

## Introduction

Somatic plant cells retain their developmental capabilities and under appropriate conditions can dedifferentiated (i.e., assume stem cell like state) and give rise to different cell types that make up a new fertile plant. In plants, dedifferentiation characterizes the transition of differentiated leaf cells to protoplasts (plant cells devoid of cell walls), which is accompanied by widespread chromatin decondensation [1–3], a feature characterizing stem cells both in plants and animals [4,5]. Interestingly, somatic nuclei derived from chicken erythrocytes were induced to dedifferentiate by *Xenopus* egg extract, which was accompanied by prompt chromatin decondensation [6]. This suggests that besides epigenetic means other mechanisms might be involved to bring about prompt acquisition of decondensed chromatin state. Thus, we assumed that one way by which condensed chromatin can assume a relaxed state is by nicks or double strand DNA breaks (DSBs) being introduced into superhelical DNA by the activity of S1-type single-stranded DNA endonucleases. Torsional strain generated in superhelical DNA, which is common in condensed chromatin often leads to local denaturation and unpairing [7], which allows single-stranded DNA endonucleases to generate nicks and/or DSBs to bring about chromatin relaxation. This is well demonstrated by the conversion of supercoiled plasmid DNA into relaxed and linear forms by S1-type endonucleases [8,9].

S1-type endonucleases are the main nucleases implicated in DNA degradation that occurs during programmed cell death (PCD) in plants. Plant endonucleases are divided into two classes based on their requirement for divalent cations for activity, namely, Zn^2+^-dependent endonucleases and Ca^2+^-dependent endonucleases [10] (Sugiyama et al., 2000). They are commonly bifunctional enzymes that can efficiently degrade single stranded DNAs (ssDNAs) and RNAs but they are almost not active toward double stranded DNA (dsDNA, [11–13]. S1/P1-type endonucleases were isolated from a variety of plant species [10] and based on their amino acid sequence they were classified as orthologs of the well known fungal S1 and P1 nucleases from *Aspergillus oryzae* and *Penicillium citrinum*, respectively [14]. An extensive work was done to reveal their enzymatic properties including requirement for divalent cations, pH optimum and substrate specificity [10,15]. *Arabidopsis thaliana* contains five genes encoding for putative S1-type endonucleases named ENDO1 to 5 [16]. Based on amino acid sequence alignment and phylogenetic tree, the ENDO proteins were divided into three groups, which composed of ENDO1/BFN1 (At1g11190) in group I, ENDO3 (At4g21590), ENDO4 (At4g21585) and ENDO5 (At4g21600) in group II and ENDO2 (AT1G68290) in group III [16]. These endonucleases display variability in requirements for divalent cations and optimum pH for their catalytic activity [15]. Consequently, while ENDO1 and ENDO2 displayed strong activity toward single stranded DNA (ssDNA) in the presence of Ca^2+^ and Mn^2+^ at pH 8.0, ENDO3 had high activity toward ssDNA in the presence of Zn^2+^ and at pH 5.5. Structure modeling of the Arabidopsis endonucleases revealed that all nucleases have similar folding as the P1 nuclease despite a relatively low amino acid sequence similarity [15]. Also the orientations of amino acids within the predicted zinc-binding domain at the active sites of all *Arabidopsis* endonucleases appear to be similar with the exception of ENDO2 whose structure modeling showed different orientation of ASP45 and LYS48 [15]. Indeed, ENDO2 was found to be a more versatile enzyme than the other endonucleases as it can degrade ssDNA and dsDNA in the presence of Zn^2+^ at acidic pH of 5.5 [15].

The Arabidopsis ENDO1/BFN1 is the best-studied S1-type endonuclease, whose expression is associated with senescence and PCD [17]. Indeed, BFN1 expression is induced during leaf and stem senescence and BFN1 promoter directed the expression of a GUS reporter gene in senescing leaves, abscission zone of flowers and xylem as well as in developing anthers and seeds, and in floral organs after fertilization [18]. In transient expression assays, BFN1, initially displayed ER localization in tobacco protoplasts, but as they senesced BFN1 could not be observed inside nuclei but rather appeared clustered around nuclei; in transgenic *Arabidopsis* plants ENDO1 was found associated with fragmented nuclei in membrane-wrapped vesicles [19]. It appears that BFN1/ENDO1 is involved in various processes associated with cell death [20,21]. The differences in amino acid sequence as well as requirements for pH and divalent cations for their activities suggest that these enzymes either perform different functions in plant growth and development or expressed at different developmental stages and different plant organs. The search of available microarray databases using the GENEVESTIGATOR platform [22] revealed that ENDO5 is restrictively expressed in root cells, ENDO1 in stamen and certain root tissues and cells including xylem, epidermis and root protoplasts, ENDO3 is expressed mainly in flower organs, while ENDO2 is expressed in leaf cell protoplasts as well as in response to various biotic and abiotic stresses (Fig A & Fig B in S1 Appendix). However, the role played by ENDO2 in plant growth and development as well as in PCD is largely unknown. Here we showed that transition from leaf cells to protoplasts is accompanied by prompt, gradual chromocentric chromatin decondensation, which is accompanied by abrupt, transient increase in endonuclease activity corresponding to ENDO2. ENDO2 fused to GFP showed a dynamic subcellular distribution being cytoplasmic in healthy protoplasts and nuclear in non-healthy, senescing protoplasts where ENDO2 is associated with fragmented nuclei. Mutation of the *ENDO2* gene did not block prompt chromatin decondensation. Our data do not support a role for ENDO2 in abrupt chromatin decondensation upon protoplasting but rather suggest a role for ENDO2 in early stages of PCD, that is, fragmentation of the nucleus.

## Materials and Methods

### Plant growth conditions

Seeds of Arabidopsis (Col) wild type and *endo2* mutant (SALK_130325, obtained from ABRC) were sown and incubated at 4°C for 4 days (stratification) after which plants were grown under long-day conditions (16 h light) at 23°C.

### Construction of plasmids

We generated two constructs of ENDO2 in which a polyhistidine (6xHis) was linked either to the N terminus of to the C terminus. To generate pUC19-35S-His-ENDO2-GFP, the ENDO2 cDNA was cloned by PCR using cDNA prepared on total RNA isolated from protoplasts as template and the following primers, His-ENDO2-S flanked with *Bam*HI restriction site and codons for six histidines 5’- CACAGGATCCATGCACCATCACCATCACCATATGGCAAACCAAAAAGGGTTACATG and ENDO2-AS flanked with *Sma*I restriction site 5’-TGTGCTCGAGTCACCCGGGACCGAAAATCCTGTTTAGG. The PCR product was digested with *Bam*HI and *Sma*I and subcloned into *Bgl*II and *Sma*I sites of pUC19 downstream from the 35S promoter and in frame with GFP. The pUC19-35S-ENDO2-His was generated by PCR using the following primers, ENDO2-S 5’-ACACGGATCCATGGCAAACCAAAAAGGGTTACATGTAG and ENDO2-His-AS 5’–TGTGCCCGGGTCAATGGTGATGGTGATGGTGACCGAAAATCCTGTTTAGGGTAGC. The PCR product was then digested with *Bam*HI and *Sma*I and subcloned into *Bgl*II and *Sma*I sites of pUC19 downstream from the 35S promoter. All constructs were confirmed by sequencing. The endoplasmic reticulum marker calnexin fused to GFP (pBI221-CNX-GFP) was kindly provided by Dr. Liwen Jiang [23].

### RNA extraction and analysis

RNA was prepared from Arabidopsis leaves and protoplasts using RNeasy Plant Mini Kit (Qiagen) according to the manufacturer’s protocol. 1 μg of total RNA was used for cDNA production using the Verso cDNA kit (Thermo scientific) and oligo dT as a primer. The resulting cDNA was subjected to PCR to amplify the ENDO2 cDNA sequence using ENDO2-RT-F 5’-CACTCAAAAAGAATATCACGACAG and ENDO2-RT-R 5’-GTGTTCGGGGCTTGTGATTG. The Ubiquitin10 (At4g05320) sequence was used as a control with the following primers: UBQ10-F 5’-TTTGTTAAGACTCTCACCGGAAAGACA and UBQ10-R 5’-GAGGGTGGATTCCTTCTGGATATTGTA. We used GoTaq Green Master Mix (Promega) and PCR conditions were 94°C, 5 min; 30–35 cycles of 94°C, 30 s; 56°C, 30 s; 72°C, 30 s; followed by 72°C, 5 min. PCR products were resolved on 1% agarose gel stained with ethidium bromide.

### Protoplasts preparation and transient transformation

Transient expression in protoplasts was performed essentially as described [24]. *Arabidopsis* fresh leaves were cut into small pieces, incubated in a cell wall degrading solution containing 1–1.5% cellulase, 0.3–0.5% macerozyme, 0.4M mannitol, 20mM KCl, 20mM MES, 10mM CaCl_2_ and 0.1% BSA, placed in a vacuum for 20 minutes, and then shaken for 90–120 minutes at 50 rpm. The protoplasts were then filtered through a 180 μm mesh, diluted with 1 volume of W5 (150mM NaCl, 125mM CaCl_2_, 5mM KCl and 2mM MES) and pelleted by centrifugation (Room temperature, 2 min at 300 x g). The protoplasts were re-suspended in W5 solution and incubated for 30 minutes on ice, before being centrifuged again and re-suspended in 100μl of MMg solution containing, 0.4 M mannitol and 15 mM MgCl_2_. Appropriate plasmid DNA (5–20 μg) was added to protoplasts and equal volume of 40% PEG solution (in 0.2M mannitol and 0.1 M CaCl_2_) and the mixture was incubated for 15–30 minutes. Two volumes of W5 were added to each sample, centrifuged for two minutes, re-suspended in 1ml of W5 and then incubated in a growth room at 22°C±2 in the dark for 24–96 h. Protoplasts were inspected at various time points after transformation by a confocal microscope (Zeiss Meta510).

### Nuclei preparation and FISH assay

Nuclei were prepared from leaves and protoplasts as previously described [2]. FISH analysis was performed according to Avivi et al. [25]. Briefly, fixed nuclei (5–10 ml, kept at -20°C in ethanol:acetic acid/3:1) were spread on a slide, air-dried, and incubated in 100% ethanol for 1 hr at room temperature. Slides were then air-dried and incubated for 6 min in a fixative solution containing freshly prepared 4% paraformaldehyde in 1x SSC. Slides were washed three times, 5 min each, with 2x SSC and subjected to denaturation solution containing 70% formamide in 1x SSC at 60°C for 3 min followed by sequential washes, 3 min each, in 70, 95, and 100% cold ethanol. Slides were air-dried and used for hybridization. BACs [kindly provided by the *Arabidopsis* Biological Resource Center (ABRC)] F2H10, T10P12, F2J6, and F28H19 (Chr 1) were labeled with tetramethylrhodamine-5-dUTP using the Nick translation reaction essentially as described [26]. CEN180 was labeled by PCR using fluorescein-12-dUTP (Roche) and the following primers: 180-S 5’-GAGAGGATCCCGTAAGAATTGTATCCTTGTTAG and 180-AS 5’-GAGAGAATTCCCTTTAAGATCCGGTTGTGG. Each probe was mixed with a hybridization solution (final volume 100 μl) containing 10% (w/v) sodium dextran sulphate, 50% deionized formamide and 2x SSPE, denatured at 90°C for 5 min, and cooled on ice. Probes were added to slides, covered with a cover glass, and incubated at 37°C in the dark for 16–20 hr followed by washings as described [27]. Slides were then stained for 10 min with 10 μg/ml diamidinophenylindole (DAPI), washed twice, and mounted in Vectashield (Vector Laboratories, CA), and inspected under a confocal microscope (Zeiss LSM 510 META).

### Protein extraction, DNA endonuclease activity, in gel nuclease assay and Concanavalin A-agarose chromatography

Leaves or protoplasts were homogenized in NETN buffer (100 mM NaCl, 1 mM EDTA, 20 mM Tris, pH 8, and 0.5% NP-40) containing protease inhibitors (sigma plant protease inhibitor cocktail at 1:100 dilution). Homogenates were centrifuged (15 min, 12,000xg, 4°C), the supernatant was collected, aliquoted and stored at -80°C until used. Protein concentration was determined with the Bradford reagent (BioRad). Nicking and single strand endonuclease activities were analyzed by the conversion of supercoiled plasmid DNA into relaxed and linear forms essentially as described [9].

In gel nuclease assays were performed essentially as described [28] in polyacrylamide gel containing 300 μg/ml denatured salmon sperm DNA or ribonucleic acid from torula yeast (Sigma) for RNases activity. Briefy, 10 μg proteins extracted from various tissues (indicated in the text) were incubated with sample buffer containing 2% SDS, 62.5mM Tris pH 6.8 and 10% glycerol and bromophenol blue for 1h at 37°C followed by separation on SDS/PAGE (samples were not boiled). The gel was washed twice, each time for 30 min, at room temp in buffer containing 10 mM Tris-HCl pH 7.5 and 25% isopropanol, followed by washing twice 15 min each with 10 mM Tris-HCl pH 7.5. Nuclease activity was performed by incubating the gel with 10 mM Tris-HCl pH 7.5 containing divalent cations (10 mM MgSO_4_, 10 mM CaCl_2_) for 75 min at 37°C. The gel was stained for 60–80 minutes with 10 mM Tris HCl pH 7.5 containing 2 μg/ml ethidium bromide and inspected under UV light. Purification of glycoproteins was performed on ConA-agarose (Sigma). Total proteins extracted from Arabidopsis leaves or protoplasts were loaded on ConA-agarose column in PBS buffer containing 500 mM NaCl and eluted by 10 mM Tris pH 7.5 and 500 mM mannose.

## Results

### Chromocentric chromatin undergoes prompt decondensation upon protoplasting

**Fig 1:**
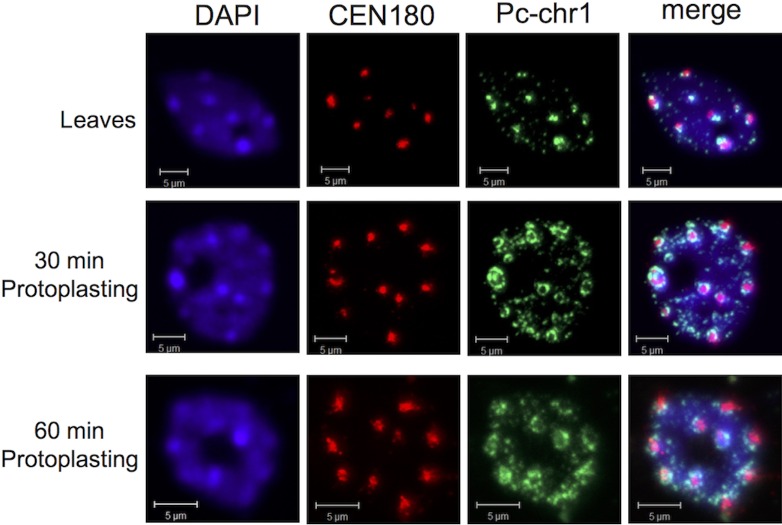
Chromocentric chromatin undergoes prompt and gradual decondensation upon protoplasting. Nuclei prepared from *Arabidopsis* leaves (cont) or from leaves treated for 30 and 60 min with cell wall degrading enzymes were subjected to FISH using fluorescein-labeled PC-Chr1 (pericentric region) and rhodamine-labeled CEN180. Note chromatin decondensation is gradual initiating at the pericentromer followed by decondensation of the centromeric chromatin. DAPI was used as a counterstain. Bar = 5 μm.

Previous work in our lab and others have demonstrated that heterochromatic chromosomal sites, namely centromeric and pericentromeric regions assume an open conformation when analyzed 12 to 16 h following protoplasting [2,3]. However, reported data in animals showed that chromatin decondensation can occur promptly within a few minutes when erythrocyte nuclei are incubated in Xenopus egg extract [6]. We sought to determine how fast chromocentric regions assume decondensed configuration following protoplasting, that is, the application of cell wall degrading enzymes (cellulose). Fluorescence in situ hybridization (FISH) using both centromeric repeats (CEN180-rhodamine) and pericentromeric (PC-Chr1-fluorescein) probes [25] revealed that the chromocenter region undergoes gradual decondensation following treatment with cell wall degrading enzymes (Fig 1). The results clearly showed that the pericentric region is the first to undergo decondensation (after 30 min) followed by decondensation of the centromeric region (CEN180), which is evident after 60 min of protoplasting. We hypothesized that the prompt opening of chromatin is unlikely to be driven solely by epigenetic means and that other factors such as S1-type endonucleases might be involved to bring about immediate and direct chromatin de-compaction.

### Endonuclease activity is upregulated in dedifferentiating protoplasts

**Fig 2:**
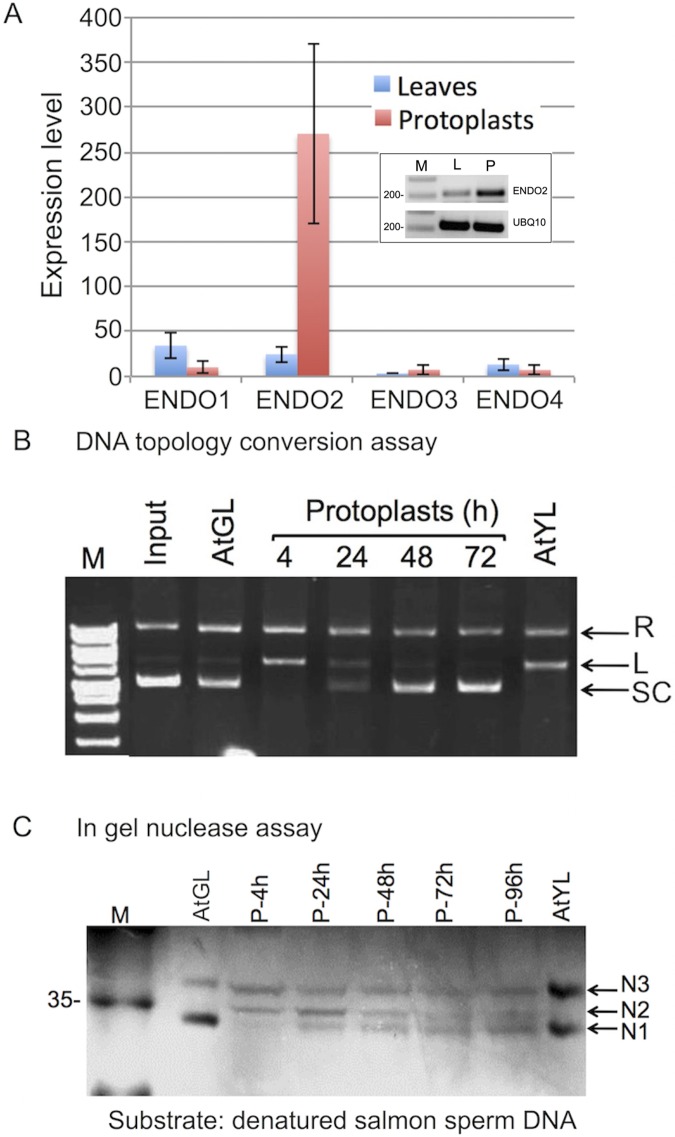
Endonuclease activity is enhanced in protoplasts immediately after preparation. (**A**) *ENDO2* is up-regulated in protoplasts. Based on transcriptome profiling of protoplasts obtained following 12–14 h incubation of *Arabidopsis* leaves with cell wall degrading enzymes [29]. Bars represent standard deviation. Inset shows RT-PCR demonstrating low and high expression of ENDO2 in leaves (L) and protoplasts (P), respectively. UBQ10 was used as a reference RNA. M, DNA size markers. (**B**) *In vitro* endonuclease conversion assay using proteins (3 μg) extracted from isolated protoplasts at various time points after preparation. Supercoiled plasmid DNA (Input, 1 μg) was incubated for 30 seconds at room temperature and DNA topology was analyzed by agarose gel electrophoresis. The positions of the different topological forms of plasmid DNA are indicated: R, relaxed, L, linear, SC, supercoiled plasmid DNA. Input is 1 μg of supercoiled plasmid DNA. Endonuclease activity in proteins extracted from green (AtGL) and yellow (AtYL) leaves was used as a reference. Note that endonuclease activity in protoplasts immediately after preparation (4h) is comparable to that of yellow, senescing leaves. (**C**) In-gel nuclease assay. Proteins (30μg) extracted from *Arabidopsis* green (AtGL) and yellow (AtYL) leaves or from protoplasts at the indicated time points after preparation were separated on polyacrylamide gel containing denatured salmon sperm DNA and subjected to in gel assay. N1, N2 and N3 nucleases are indicated by arrows.

Transcriptome profiling of dedifferentiating protoplasts [29] revealed that among the four S1-type endonucleases present on the affymetrix ATH1 array only ENDO2 was upregulated in protoplasts (Fig 2A). The upregulation of ENDO2 in protoplasts was confirmed by RT-PCR (inset Fig 2A). The occurrence of S1-type endonuclease activity in protoplasts was studied by using the plasmid DNA topology conversion assay (Fig 2B), that is, the conversion of supercoiled (SC) plasmid DNA into relaxed and linear forms–an established method for monitoring single strand DNA endonucleases [8]. Proteins extracted from *Arabidopsis* protoplasts at various time points after isolation were incubated for 30 second at room temperature with 1 μg of essentially supercoiled plasmid DNA. Reactions were stopped by adding EDTA (50 mM) and samples were run on 1% agarose gel containing ethidium bromide. A notable S1-type endonuclease activity was recovered in protein extract derived from protoplasts 4 h after isolation as demonstrated by the conversion of supercoiled plasmid DNA into relaxed and linear forms. This activity was gradually diminished in protoplasts so that after 24 h slight activity was still evident but no activity could be detected after 48 and 72 h (Fig 2B). Notably, very low activity could be detected in protein extracts derived from green leaves of Arabidopsis, while yellow, senescing leaves display endonuclease activity comparable to that of 4h protoplasts.

Next we performed in gel nuclease assay to monitor nuclease activity in leaves and protoplasts. Proteins extracted from leaves and protoplasts at various time points after preparation were subjected to in-gel nuclease assay using denatured salmon sperm DNA as substrate. Results showed that green leaves (AtGL) as well as yellow leaves (AtYL) of Arabidopsis displayed two protein bands of nuclease activity at positions of about 34 kDa (designated N1) and ~38 kDa (designated N3) (Fig 2C). In yellow leaves, N3 nuclease activity was much higher than its activity in green leaves. Interestingly, immediately after protoplasts preparation (4h) N1 activity disappeared and a new slow migrating nuclease designated N2 at position of about 36 kDa was apparent. N2 nuclease activity was increased at 24 h and then gradually reduced concomitantly with the recovery of N1 nuclease, implying that N1 and N2 are the products of the same gene. N3 nuclease activity in protoplasts remained essentially unchanged at all stages of protoplast development.

### N1 and N2 nucleases are the products of the *ENDO2* gene: analysis of *endo2* mutant

**Fig 3:**
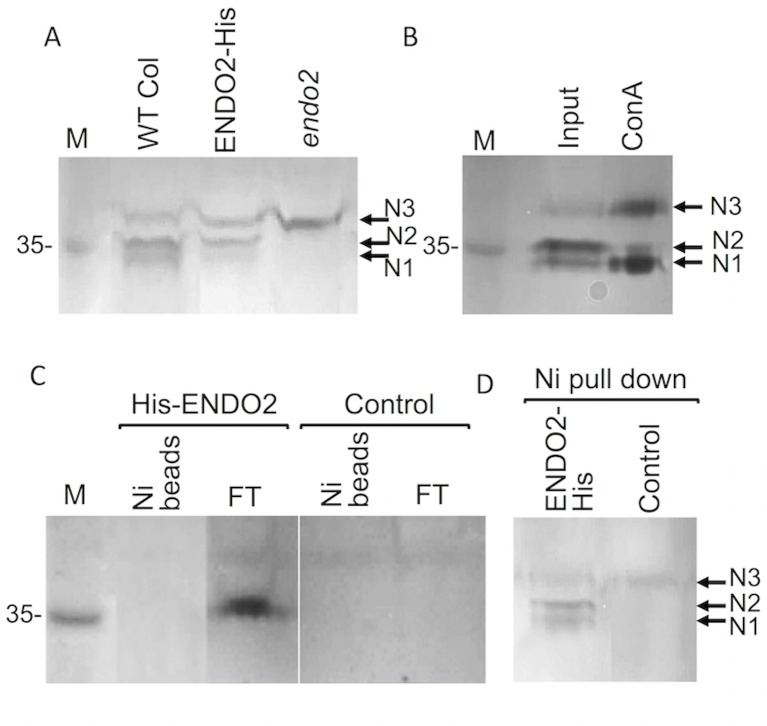
ENDO2 is modified in protoplasts. (A) N1 and N2 nucleases are the product of the *ENDO2* gene. In gel nuclease assay of proteins extracted from protoplasts derived from WT leaves (WT Col), *endo2* mutant leaves and from *endo2* protoplasts transformed with pUC19-S35-ENDO2-His. Note that *endo2* protoplasts lack N1 and N2 nucleases. (B) ENDO2 N2 variant is not glycosylated. Proteins extracted from WT protoplasts (24h) were loaded on ConA-agarose and bound proteins were subjected to in gel nuclease assay. Note that ConA enriched the N1 and N3 nucleases. (C) ENDO2 is cleaved at its N terminus. ENDO2 was tagged with histidine (6X) at its N terminus (His-ENDO2) and the plasmid pUC19-35S-His-ENDO2 was transformed into *endo2* protoplasts. His-tagged proteins extracted were purified on nickel column and bound proteins (Ni beads) as well as the flow through (FT) fraction were subjected to in gel assay. Control indicates untransformed *endo2* protoplasts. (D) ENDO2 is not cleaves at its C terminus. ENDO2 was tagged with histidine (6X) at its C terminus (ENDO2-His) and the plasmid pUC19-35S-ENDO2-His was transformed into *endo2* protoplasts. Bound proteins were subjected to in gel assay. Control indicates untransformed *endo2* protoplasts.

As mentioned above, *Arabidopsis thaliana* contains five genes encoding for putative S1-type endonucleases named ENDO1 to 5 [16]. The microarray database of Arabidopsis leaves versus protoplasts [29] showed that among the four endonucleases present on the affymetrix ATH1 array only ENDO2 is upregulated in protoplasts (Fig 2A). To examine whether N1 and N2 are the products of the *ENDO2* gene we obtained from ABRC a SALK T-DNA homozygous knockout line (SALK_130325) for the *ENDO2* gene and tested by in gel assay nuclease activities in *endo2* protoplasts. Results showed (Fig 3A) that in contrast to wild type, *endo2* protoplasts are lacking activity corresponding to N1 and N2 nucleases, suggesting that both activities are related to ENDO2 protein. The ENDO2 nuclease activity in *endo2* mutant protoplasts is recovered following transient expression of ENDO2 construct (using pUC19-S35-ENDO2) displaying nuclease activity at the expected sizes of about 35 kDa (Fig 3A).

### ENDO2 cleaved at its N-terminus and its variants are differentially glycosylated

We next wanted to gain insight into the nature of the ENDO2-N1 and ENDO2-N2 variants assuming that N1 and N2 represent differentially glycosylated forms. Endonucleases are known to be modified by N-glycosylation, which is often required for their activity. ENDO2 contains two N-glycosylation sites required for activity [30], which appear to be conserved among plant endonucleases [13]. We used Concanavalin A (ConA)-agarose to pull down glycoproteins from protein extracts derived from protoplasts 24 h after isolation, which were eluted with mannose followed by in-gel nuclease assay. Results showed (Fig 3B) that N1 and N3 nucleases are glycosylated as they could be enriched by Con A, while N2 nuclease was only slightly recovered by ConA, suggesting that N2 is either not glycosylated or being decorated with sugars which do not bind ConA. Notably, ENDO2 was previously shown to undergo cleavage of 27 amino acids from its N terminus in transgenic plants [15,30]. We wanted to investigate if cleavage of ENDO2 at its N terminus can occur in protoplasts. To this end, we generated a fusion protein in which six histidines (6XHis) were fused to the N terminus of ENDO2 and placed under the control of the 35S promoter followed by transient expression into *endo2* protoplasts. After 24 h incubation, proteins were extracted, loaded onto Nickel (Ni)-agarose beads commonly used to purify polyhistidine-tagged proteins and bound proteins were analyzed by in-gel nuclease assay. A control was done using the same procedure, but without adding the His-ENDO2 construct. The in-gel assay showed (Fig 3C) that the Ni beads did not pull down His-ENDO2 protein, which was recovered in the flow through (FT) fraction suggesting that His-ENDO2 was cleaved at its N terminus to remove the signal peptide consistent with previous reports [15,30]. Finally, we tested the possibility that ENDO2 may undergo cleavage also at its C-terminal region. A recent report suggested that the C-terminal end of the Arabidopsis ENDO2 may be proteolytically cleaved though un-efficiently [15]. To assess this possibility we tagged the C-terminal end of ENDO2 with 6XHis and transformed into protoplasts. Transformed protoplasts were analyzed 24 h after transformation for ENDO2-His expression by loading protein extracts onto Ni beads followed by separation on SDS/PAGE and in gel nuclease assay. Results showed (Fig 3D) that the C terminus is not cleaved significantly inasmuch as ENDO2-His can be recovered by Ni beads from extracts derived from protoplasts 24 h after transformation.

### ENDO2-GFP is cytoplasmic in healthy protoplasts but associates with fragmented nuclei in senescing/dying protoplast cells

**Fig 4:**
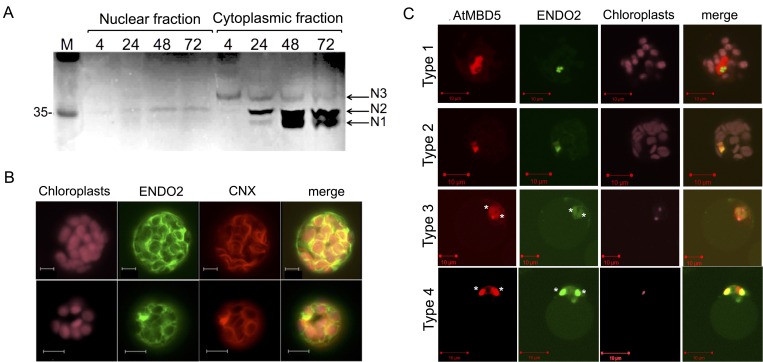
ENDO2 is essentially cytoplasmic in healthy cells but translocates to the nucleus in senescing, unhealthy cells. (A) Most nuclease activity resides in the cytoplasm and a small fraction of ENDO2-N2 variant is found in the nucleus. Cytoplasmic and nuclear fractions were prepared from protoplasts at various time points after preparation and subjected to in gel nuclease assay. (B) ENDO2-GFP is associated with the ER in healthy cells. Protoplasts derived from WT leaves were cotransformed with ENDO2-GFP and ER marker CNX-RFP and analyzed under a confocal microscope after 24 h. (C) ENDO2 display various localization patterns in the nucleus of aging protoplasts. Wild type protoplasts were co-transformed with pS35-ENDO2-GFP and pS35-AtMBD5-RFP and inspected at various time points after transformation. Note the various types of ENDO2 nuclear localization and ENDO2 association with fragmented nuclei (marked with asterisks) in senescing, dying protoplasts (Type 3 and 4).

S1-type endonucleases are capable of degrading both RNA and single-stranded DNA and might play a role in RNA and DNA metabolism. Analysis of the ENDO2 protein sequence using cNLS mapper (for prediction of importin α-dependent nuclear localization signals; http://nls-mapper.iab.keio.ac.jp/cgi-bin/NLS_Mapper_form.cgi) revealed a bipartite NLS with a score of 5.3 suggesting that the ENDO2 protein is localized to both the nucleus and the cytoplasm. To verify this, we obtained total proteins from protoplast cytoplasmic fraction or from the nuclear fraction and analyzed for nuclease activity using in-gel assay. The results showed (Fig 4A) that only a small fraction of N2 nuclease is found in the nucleus, yet most nuclease activities (N1, N2 and N3) are found in the cytoplasm.

To visualize ENDO2 subcellular localization, we amplified by PCR *ENDO2* cDNA and cloned it downstream from the 35S promoter and in frame with GFP to generate pUC19-35S-ENDO2-GFP construct. This construct was transformed into protoplasts prepared from WT plants and inspected under a confocal microscope. Results showed (Fig 4B) that in healthy protoplasts ENDO2-GFP is essentially localized in the cytoplasm surrounding chloroplasts in a speckle-like or thread-like distribution, co-localized with the ER marker CNX-RFP. This localization pattern reminiscent that of wheat S1-type endonuclease TaS1L-GFP in *Arabidopsis* protoplasts [13] as well as of BFN1/ENDO1 in tobacco protoplasts [19]. To investigate localization of ENDO2-GFP in the nucleus, we co-transformed protoplasts with ENDO2-GFP and AtMBD5-RFP, a nuclear-localized protein [31]. Inspection under a confocal microscope revealed various patterns of localization of ENDO2-GFP within the nucleus. Some protoplasts, inspected 2 days after transformation, showed ENDO2-GFP in the nucleus exclusively localized in nucleoli (Fig 4C, Type 1). Later, a higher fraction of protoplasts showed that ENDO2-GFP is dispersed throughout the nucleus co-localized with AtMBD5-RFP (Fig 4C, Type 2). In unhealthy, senescing protoplasts (cytoplasm shrinkage and aggregated or degraded chloroplasts) ENDO2-GFP is often associated with fragmented nuclei, co-localized with AtMBD5-RFP (Fig 4C, Type 3 and 4).

### Mutation in *ENDO2* gene does not block chromocentric chromatin decondensation

**Fig 5:**
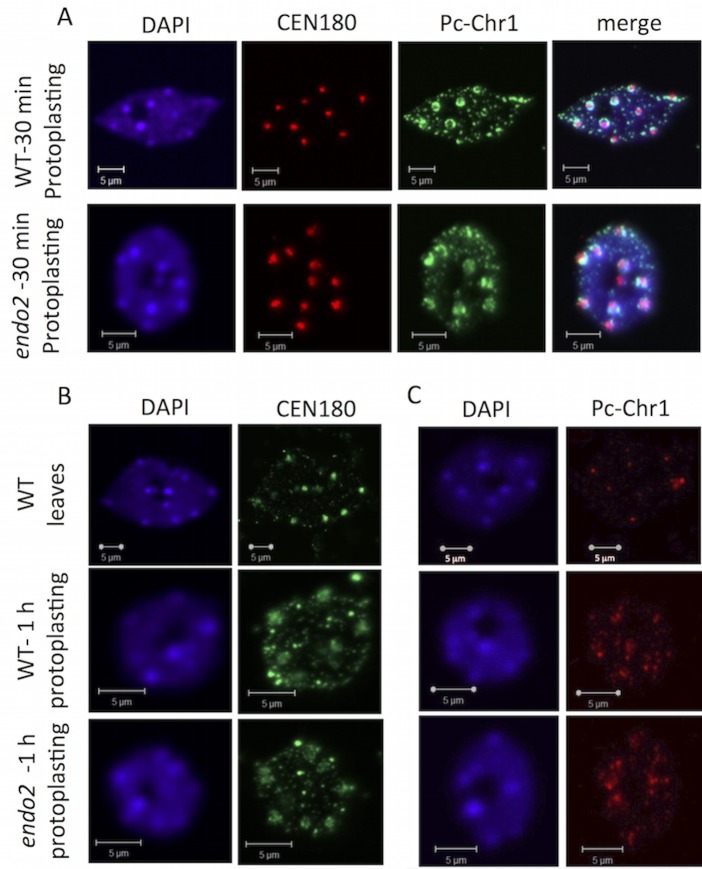
Mutation of the *ENDO2* gene does not block prompt chromocentric chromatin decondensation upon protoplasting. (A) Nuclei prepared from protoplasts derived from WT and *endo2* leaves treated for 30 min with cell wall degrading enzymes were subjected to FISH using rhodamine-labeled CEN180 and fluorescein-labeled PC-Chr1. (B, C) Nuclei prepared from WT leaves or from protoplasts derived from WT and *endo2* leaves treated for 1 h with cell wall degrading enzymes were subjected to FISH using fluorescein-labeled CEN180 (B) and and rhodamine-labeled PC-Chr1 (C). DAPI was used as a counter stain.

We next analyzed by FISH the effect of mutation in the *ENDO2* gene on chromocentric chromatin conformation upon protoplasting. Nuclei prepared from protoplasts derived from WT and *endo2* leaves treated for 30 (Fig 5A) or 60 min (Fig 5B and 5C) with cell wall degrading enzymes were subjected to FISH with either fluorescein-labeled centromeric 180 bp repeats (CEN180) or rhodamine-labeled PC-Chr1. Results showed that chromocentric chromatin decondensation in *endo2* protoplasts was similar to that of WT protoplasts. Both showed prompt and gradual decondensation of chromocentric chromatin suggesting that ENDO2 is not required for the prompt decondensation of chromocentric chromatin that occurs upon protoplasting.

## Discussion

We showed here that chromocentric regions decondensed their chromatin promptly but gradually with pericentric decondensation occurring first within 30 min, and slightly later within 60 min, the centric region assumed open chromatin conformation. We also showed that chromatin decondensation was associated with increase in S1-type endonuclease activity driven by ENDO2, which was gradually diminished thereafter. However, we could not show a linkage between prompt chromatin decondensation and ENDO2 activity inasmuch as mutation of the ENDO2 gene did not block or delay chromocentric chromatin relaxation. Using *endo2* mutant plants and in gel nuclease assays we found that the nuclease activities in protoplasts, designated N1 and N2 are contributed by ENDO2. Interestingly, ENDO2 displayed a peculiar localization in protoplasts, that is, cytoplasmic in healthy cells, often showed distinct localization within nucleoli; in senescing protoplast cells ENDO2-GFP showed association with fragmented nuclei. This mode of localization is similar to that reported for wheat endonuclease TaS1L in *Arabidopsis* protoplasts [13]. Also, transient expression of BFN1/ENDO1 in tobacco protoplast cells showed cytoplasmic, ER-associated localization in normal tobacco protoplasts but localization around nuclei in senescing protoplasts [19]. In transgenic *Arabidopsis* plants expressing BFN1-GFP, the protein was colocalized with fragmented nuclei in membrane-wrapped vesicles in late senescence [19]. Furthermore, we showed that the cytoplasmic variant of ENDO2 (N1) is glycosylated and capable to bind ConA, while the nuclear variant (N2) is essentially unglycosylated or is decorated with ConA non-binding sugars.

The finding that most ENDO2 in healthy protoplasts showed cytoplasmic, ER localization raises a question regarding its function in the cytoplasm. Commonly, endonucleases have been implicated in DNA metabolism being particularly involved in DNA repair [32]. In plants, endonucleases were studied with respect to programmed cell death (PCD) that occur during development including leaf senescence, endosperm degeneration and tracheary element differentiation as well as in hypersensitive response to infection by pathogens [10]. Yet, the dwelling of endonucleases such as BFN1/ENDO1, ENDO2 and TaS1L in the cytoplasm often associated with the ER [13,19] implies for a role in the cytoplasm. One possible function could be related to the fact that BFN1/ENDO1 and ENDO2 are S1-type endonucleases characterized by their capability to degrade both single strand DNA and RNA [30,33] and thus might be involved in RNA metabolism, that is, RNA stability and translatability.

In protoplasts, we repeatedly observed two variants of ENDO2 proteins, which we designated N1 and N2. While N1 is efficiently recovered by ConA, N2 is not. Considering that ConA has high affinity for glycoproteins having mannose and glucose [34] it is possible that N2, which appears to be localized also in the nucleus, is mostly unglycosylated. Alternatively, glycan chain in N2 may be decorated with sugars like galactose with low affinity of ConA. The role of N-glycosylation of ENDO2 has been studied showing that it is required for stability and activity [30]. Since ENDO2, like BFN1/ENDO1 does not possess a canonical ER retention signal at its c-terminus (e.g., KDEL, HDEL) other signal might be involved in its cytoplasmic/ER retention or that ENDO2 retention in the ER is independent of a specific signal [35]. Alternatively, glycosylated ENDO2 may be retained in the ER via binding to ER molecular chaperones that function as lectins such as calnexin and calreticulin [36]. Thus it is possible that glycosylation is required for retention of ENDO2 in the ER rendering it incapable of translocation into the nucleus. Accordingly, upon induction of senescence, ENDO2 undergoes multiple modifications making it competent for translocation into the nucleus. These include de-glycosylation to release ENDO2 from the ER, which is accompanied by phosphorylation often found in animal cells to trigger translocation of cytosolic proteins into the nucleus [37,38]. Indeed, analysis of the ENDO2 protein by NetPhos 3.1 software revealed multiple potential phosphorylation sites at serine and tyrosine residues (not shown). We also found that ENDO2 is undergoing cleavage at its N terminus, which is consistent with the predicted N-terminal signal peptide and with previous reports demonstrating cleavage of the N terminus in a variety of endonucleases including ENDO2 [11,15]. However, no cleavage of the C terminus of ENDO2 could be observed. Thus we assume that ENDO2-N2 variant is undergoing as yet unknown posttranslational modifications leading to slow migration in the gel compared to N1 variant but at the same time making it competent for translocation to the nucleus.

Of particular importance are the findings that in senescing cells ENDO2 was associated with fragmented nuclei, which are not wrapped by vesicles. Notably, BFN1/ENDO1-GFP was localized with fragmented nuclei in membrane-wrapped vesicles [19]. The association of ENDO2 with fragmented nuclei implies a function for ENDO2 in the first phase of DNA degradation that occurs during PCD. Here we suggest that the major function of ENDO2 and perhaps of BFN1/ENDO1 in PCD is making nuclei competent for degradation by introducing double strand DNA breaks into bulky superhelical DNA. Considering that ENDO2 as well as BFN1/ENDO1 like other S1-type endonucleases can target ssDNA but essentially not dsDNA, the only scenario where S1-type endonucleases can target nuclear dsDNA is when the strands are locally unpaired. Indeed, torsional strain generated in superhelical DNA, which is common in condensed heterochromatin often leads to local denaturation and unpairing [7], which allows single-stranded DNA endonucleases to generate nicks or cleave the DNA and thus introducing DSBs. This might lead to the formation of large fragments of DNA or to fragmentation of the nucleus. This hypothesis is consistent with the two phases of DNA degradation hypothesis suggested previously by Sugiyama et al. [10]. Accordingly, in the first phase, dying cells display a limited degradation of nuclear DNA by nuclear Ca^2+^-dependent endonucleases (e.g., ENDO2), which might lead to nuclear fragmentation, while in the second phase, the collapse of the nucleus expose DNA for degradation by Zn^2+^-dependent endonucleases, which reside in the cytoplasm (vacuole) and perhaps other DNases that completely degrade the DNA.

## Supporting Information

S1 Appendix Expression of the *Arabidopsis* endonuclease encoding genes in various tissues and following exposure to stress. (DOCX)
