# Supplementary material for: S1-Type Endonuclease 2 in Dedifferentiating Arabidopsis Protoplasts: Translocation to the Nucleus in Senescing Protoplasts Is Associated with De-Glycosylation
Source: PLoS One. 2017 Jan 9;12(1):e0170067. doi: 10.1371/journal.pone.0170067 (PMC5222596; doi:10.1371/journal.pone.0170067)
Supplement: S1 Appendix — (DOCX) [file pone.0170067.s001.docx]

S1 Appendix: Expression of the Arabidopsis endonuclease encoding genes in various tissues and following exposure to stress.


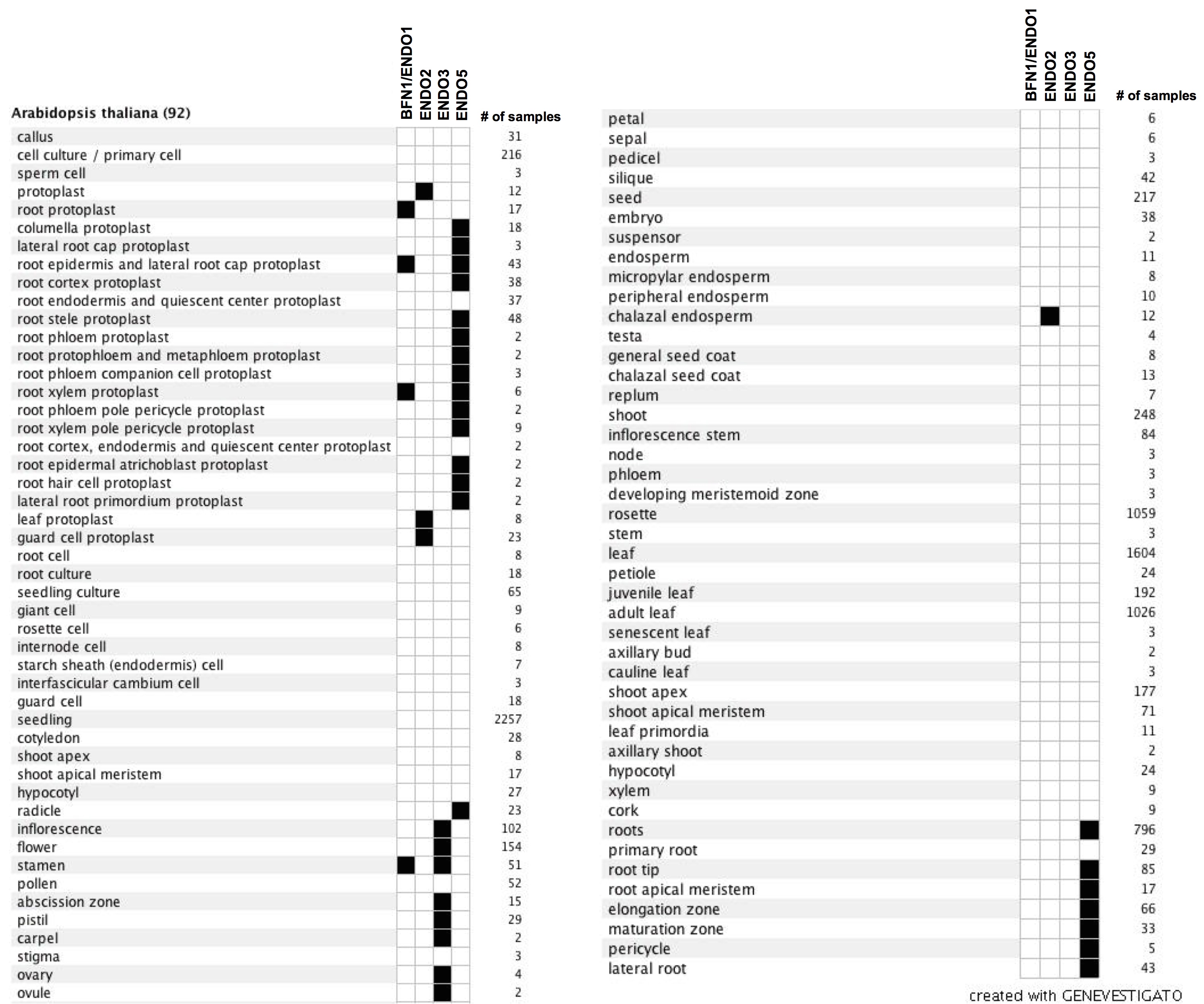
Figure A: Expression profiles of the Arabidopsis endonuclease encoding genes, *BFN1/ENDO1, ENDO2, ENDO3 and ENDO5* in various plant cells, tissues and organs. The expression ptofiles were created with Genevestigator [22].


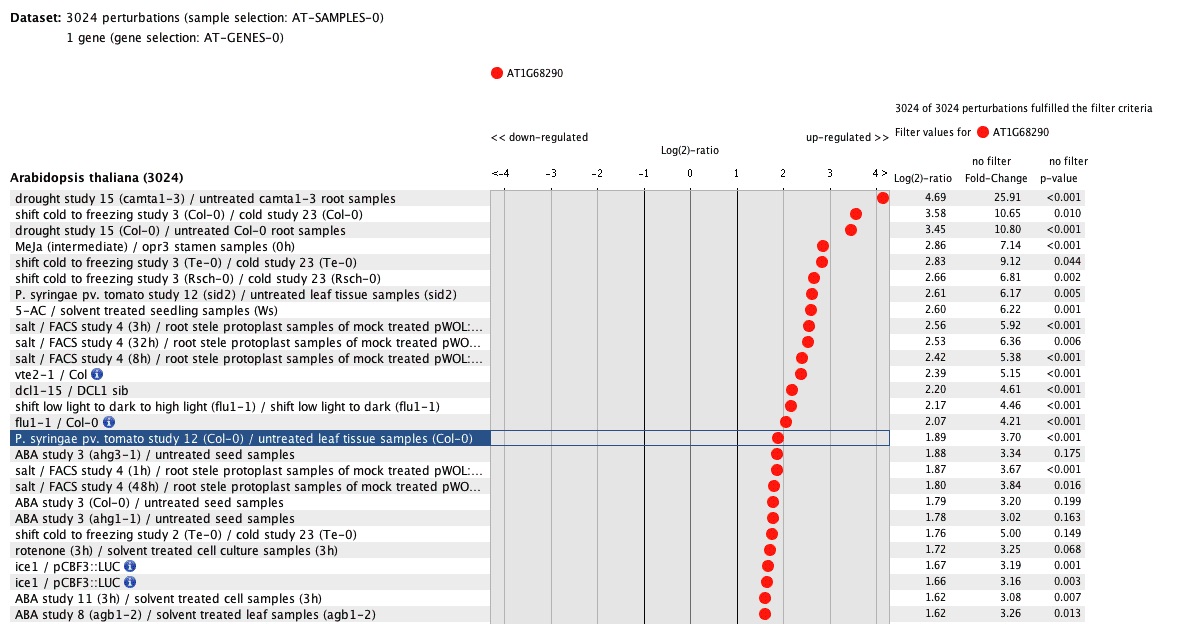


Figure B: The expression of *ENDO2* gene is induced following exposure to biotic and abiotic stress conditions. Data obtained from microarray databases using the GENEVESTIGATOR platform [22].
